# Bacteria Associated with Healthcare-Associated Infections on Environmental Samples Obtained from Two Fire Departments

**DOI:** 10.3390/ijerph182211885

**Published:** 2021-11-12

**Authors:** Kelli L. Barr, Rodney X. Sturdivant, Denise N. Williams, Debra Harris

**Affiliations:** 1 Center for Global Health Infectious Disease Research, College of Public Health, University of South Florida, Tampa, FL 10921, USA; barrk@usf.edu; 2 Department of Statistical Science, Baylor University, Waco, TX 76798, USA; Rodney_Sturdivant@baylor.edu; 3 Department of Human Sciences and Design, College of Health and Human Science, Baylor University, Waco, TX 76798, USA; DeniseNWilliams-Harr@baylor.edu

**Keywords:** healthcare-associated infections, firefighter, surface contamination

## Abstract

(1) Background: Firefighters spend about 64% of their time responding to medical emergencies and providing medical care without a patient history, which can render them vulnerable to healthcare-associated infections (HAI). Infection prevention, control, and surveillance systems have been instituted at hospitals. However, the prevalence of firefighters’ exposure to HAI is unknown. The objective of this study was to document evidence of HAI on surfaces in fire stations and engines to inform disinfection procedures and identify which pathogens might contribute to occupational exposures. (2) Methods: High-touch or high-use surfaces of two fire departments were sampled during five separate occasions. One fire station from one fire department was sampled over a 4-week period, whereas four fire stations were sampled from a different fire department only once. Sampled surfaces included: entryway floor, washing machine, medical bag, back seat of engine, keyboard of reporting computer, engine console, and uniform pants. (3) Results: Multiple statistical models determined that bacterial contamination was similar between the two fire departments and their stations. Keyboards were the most contaminated surface for all fire stations and departments, *E. coli* was the most common bacteria detected, and *C. difficile* was the least detected bacteria. Adjustments for rates of contamination found that contamination rates varied between fire stations. (4) Conclusions: Comprehensive environmental sampling and clinical studies are needed to better understand occupational exposures of firefighters to HAI.

## 1. Introduction

As first responders, firefighters participate in a broad range of activities outside of fire control. Research has shown that, nationally, firefighters spend an average of 64% of their time responding to medical emergencies [1]. Thus, just like emergency medical service (EMS) personnel, firefighters have the potential for occupational exposure to blood-borne and other pathogens, which increases their risk for occupationally acquired infections [2]. Firefighters may wear uniform trousers and shirts (which may or may not be flame-resistant) or they may wear turnout gear (that provides barriers to fire, heat, and moisture) when responding to medical emergencies. Pathogens can easily contaminate these uniforms and turnouts during rescues and medical emergencies, increasing the risk of infecting the firefighter while dressed or during doffing and donning procedures. Additionally, many firefighters are paramedics and EMS-certified and may thus provide initial emergency medical treatment. Like other EMS personnel, these firefighters must care for many different types of patients, often providing initial medical care without a patient history, which can render them vulnerable to dangerous or highly infectious pathogens. The need for proper sanitation and hygiene practices is paramount for first responders with the increasing prevalence of highly infectious pathogens, such as methicillin-resistant *Staphylococcus aureus* (MRSA), vancomycin-resistant Enterococcus (VRE), *Clostridium difficile*, tuberculosis, coronavirus, and HIV [3,4,5,6,7]. However, unlike other EMS personnel, firefighters may wear the same contaminated uniform or turnouts and ride in the same vehicle for multiple calls without undergoing decontamination.

The exposure levels of firefighters to healthcare-associated infections (HAI) are unknown, even though they are typically the initial encounter in emergency patient care. HAI are of concern because they cause bloodstream, urinary, and skin infections, as well as pneumonia and colitis, which may spread between firefighters and to others they come into contact with. Infection prevention, control, and surveillance systems have been instituted at most hospitals because, in addition to patient risk, the community is at risk of drug- and multidrug-resistant bacteria, such as MRSA and strains of *E. faecalis*, *S. pneumoniae*, and *P. aeruginosa*, that are resistant to antibiotics [7,8,9,10].

Respiratory and enteric viruses such as influenza, adenovirus, and norovirus cause regular outbreaks and annual epidemics. Each year, there are 3–5 million cases of severe influenza, causing up to half a million deaths [11]. Even though a vaccine is available for influenza, its effectiveness is roughly 60% and, even more concerning, is the lack of vaccine coverage among healthcare workers. In Europe, annual influenza vaccine coverage among healthcare workers is less than 30% [12], while in the United States, vaccine coverage is roughly 77% [13]. Unfortunately, firefighters are not included under the healthcare worker umbrella and their vaccine coverage is likely much lower. Most fire departments do not require annual influenza vaccinations despite them being recommended by the National Fire Protection Association (NFPA) [14]. Additionally, a recent poll of firefighters regarding COVID-19 vaccination found that only 50% of 1300 respondents were willing to be vaccinated without an FDA-approved vaccine [15]. This highlights a disconnect between infection control measures between healthcare and fire departments.

Viruses play a significant role in HAI, as evidence shows that viral infections predispose patients to bacterial infections through the overproduction of inflammatory cytokines and dysbiosis of systemic microbiomes [16,17,18]. For instance, infection with Influenza A virus disrupts enteric microbiota, rendering the small intestine more vulnerable to *Salmonella Typhimurium* [18]. Recent work has found that patients critically ill with COVID-19 had secondary *P. aeruginosa* and Enterobacterales infections [19]. Further, patients with norovirus, adenovirus, astrovirus, and sapovirus have increased severity of *C. difficile* symptoms [20,21].

First responders are the primary vectors for pathogens in pre-hospital settings [22,23]. Even with proper training for hand washing and use of personal protective equipment (PPE), there is often lack of compliance [24,25,26]. Lack of compliance is not intentional. In cities and other heavily populated areas, there are short periods of time between calls and a lack of time for proper cleaning of gear, PPE, and vehicles [27,28]. Uniforms [27,29], equipment [30], and vehicles [23,31,32] can harbor contamination, resulting in unintentional infection of first responders, patients, and other workers, as well as contamination of work and living environments in fire stations [5].

Bacteria, yeasts, and molds are also problematic for firefighters and other first responders, and surface contamination with these pathogens has recently been found to be significant in the transmission of many diseases [33,34,35,36,37,38,39,40,41,42,43]. A study evaluating 65 high-touch surfaces in 11 vehicles (ambulances and fire engines) and common areas of two fire stations found that bacterial, yeast, and mold contamination of the surfaces was significant [44]. Reports on surface contamination of ambulances and occupational exposures of EMS show that several drug-resistant bacteria and highly contagious viruses are of concern, especially in iatrogenic and nosocomial infections (Table 1) [44,45,46,47,48]. Low concentrations of enveloped respiratory viruses have been found to retain infectivity on common environmental surfaces, such as Teflon, polyvinyl chloride (PVC), ceramic tiles, glass, silicon rubber, and stainless steel [49]. People colonized or infected with these organisms shed these pathogens into their environments, contaminating surfaces at concentration levels sufficient for extended survival time periods and allowing for transfer to others through surface contact [50]. In fire stations, living and garage areas are susceptible to contamination, demonstrating the need for concerted effort in regular decontamination [50].

Disinfection standard operating protocols (SOPs) in place may not be sufficient in high-touch, high-use areas or against environmentally stable pathogens. The primary objective of this study was to document evidence of the HAI (see Table 1) on surfaces at fire stations and engines to inform disinfection procedures and identify which pathogens might contribute to occupational exposures.

## 2. Materials and Methods

Two fire departments located in the southern region of the United States provided access for environmental sampling of specific surfaces. Fire Department 1 (FD1) provided access to 1 fire station for repeated environmental sampling over 4 weeks. Fire Department 2 (FD2) provided access to 4 stations for incidental environmental sampling of the same surfaces as were sampled in FD1. The following surfaces were selected for environmental sampling because they were either high-touch or high-use: fire station entryway floor (vinyl), uniform washing machine (stainless steel), fire engine medical bag (canvas), back seat of fire engine (textile), fire station computer keyboard (acrylonitrile butadiene styrene or ABS), fire engine console (unknown), and uniform pants. Protective clothing uniforms were made from aramid fibers (98%) and carbon filament (2%). According to the CDC, the most common bacterial HAI are *Clostridium difficile*, MRSA, *E. coli*, *Enterococcus faecalis*, *Pseudomonas aeruginosa*, and *Streptococcus pneumoniae* [7]. These pathogens have been found to infect EMS workers and to contaminate a variety of surfaces in ambulance and healthcare settings (Table 1).

For surface sampling, sterile cotton gauze pads (2” × 2”) were moistened with sterile phosphate-buffered saline and then a 100 cm^2^ section of each surface was wiped. The gauze pads were then placed into 15 mL conical tubes containing 3 mL DNA/RNA Shield (Zymo Research #R1100) and heated to 80 degrees C. DNA and RNA were extracted using kits per the manufacturer’s instructions (Quick-DNA miniprep #D4300 and Quick-RNA Viral Kit #R1034, Zymo Research). RNA was converted to cDNA with High-Capacity cDNA Reverse Transcription Kit (Applied Biosystems #4368814) per instructions. RT-PCR was performed using TaqMan Gene Expression Master Mix (Applied Biosystems #4369542). Primer-probe sets for pathogens were obtained from diagnostic sets from the CDC, WHO, or from peer-reviewed studies on pathogen detection and diagnostics, as cited in Table 2. ΔCt analysis was performed and a two-fold change over background signal was used to determine if a pathogen was detected. Pathogens were not quantified and thus data on pathogen detection is considered in a binary nature; either the pathogen was detected and thus ‘present’ or it was ‘absent’.

A one-way ANOVA with a Tukey–Kramer post hoc test was used as a preliminary assessment, to determine if microbes were detected equally among the dates of collection and across station locations, and to determine if there were correlations between surface type and contamination. Due to the binary nature of the data, and the presence or absence of a pathogen for a given reading, logistic regression was performed to obtain odds ratios with Wald confidence intervals. Logistic regression models were fit for surfaces and pathogens separately (single variable, unadjusted) and then a multivariate model was fitted to the data. For interpreting the impact of EMS calls on bacterial contamination at a station, *p*-values were calculated to compare rates across stations using Fisher’s exact tests.

## 3. Results

The results for FD1 represent four discrete sampling events over time at a single fire station (Table 3). *M. tuberculosis*, *E. coli*, *E. faecalis*, *P. aeruginosa*, *C. difficile*, MRSA, and *S. pneumoniae* were each identified at least once from environmental samples during the 4-week period. None of the viral targets were detected. Statistical analysis of FD1′s pooled environmental sampling determined a lack of a statistical difference in the detection of microbes among the dates of collection (*p* = 0.191). When testing for interaction between surface type and contamination at FD1, no surface was more likely than any other surface to be contaminated (*p* = 0.068). Additionally, the incidence of a specific pathogen on a surface was not significant (*p* = 0.361) and the date of specimen collection was also not significant (*p* = 0.145).

The results for FD2 represent incidental sampling across four different fire stations on the same day (Table 4). Similar to FD1, no viral pathogens were detected, while *M. tuberculosis*, *E. coli*, *E. faecalis*, *P. aeruginosa*, MRSA, and *S. pneumoniae* were each identified at least once from environmental surface samples. *C. difficile* was not detected on any surface. Tukey–Kramer post hoc pairwise comparisons indicated that microbes were detected equally among the four fire stations (*p* = 0.9842). When testing for interaction between surface type and contamination, significant differences were found (*p* = 0.0465). More specifically, no bacterial pathogens were detected on the uniforms sampled at any FD2 station, and keyboards were significantly more likely to be contaminated (*p* ≤ 0.05) than any other surface. Additionally, the incidence of a specific pathogen on a surface was significant at FD2 (*p* = 0.0192).

Bacterial nucleic acid was detected in the visited fire stations on keyboards (*n* = 13), on floors (*n* = 10), and in washing machines (*n* = 9); in the fire engines on medical bags (*n* = 7), on consoles (*n* = 6), and on seats (*n* = 2); and on firefighter uniforms (*n* = 3) (Table 3 and Table 4). Comparisons using Tukey–Kramer HSD adjustments for multiple comparisons did not indicate significant differences in the occurrence of contamination between the surfaces, though keyboards were more likely to have bacteria detected, and uniforms, consoles, and seats were less likely to have bacteria detected (Table 5). Additionally, FD1 was more likely to have contaminated uniforms (*p* = 0.0277).

Table 6 reports *p*-values obtained from Tukey–Kramer comparisons of the detected bacteria between the fire departments. *E. faecalis* was most likely to be detected on surfaces at FD2 (seven occasions) than FD1 (one occasion). *S. pneumoniae* was more likely to be found on tested surfaces at FD1 (five occasions) than FD2 (one occasion) (*p* = 0.03907) but was not detected significantly more or less than any other bacteria detected. The detection of more *E. coli* at FD1 (nine occasions) than at FD2 (six occasions) was significant (*p* = 0.03907). *E. coli* was also significantly more likely to be detected at either FD than *M. tuberculosis* (*p* = 0.0117) and *C. difficile* (*p* = 0.0004). MRSA was detected seven times at FD1 and two times at FD2 (*p* = 0.047) but was not detected significantly more or less than any other bacteria detected (Table 6). There was no significance between either FD for the detection of *P. aeruginosa*, *E. faecalis*, *C. difficile*, or *M. tuberculosis* (Table 6).

Due to the binary nature of the data (present vs absent), logistic regression was performed to obtain odds ratios with Wald confidence intervals. Unadjusted models examined a single factor (surface or bacteria), while adjusted models included both surface and bacteria (Table 7). The factor with the highest count was chosen as the reference group (i.e., keyboard and *E. coli*) since having a large sample makes the model more stable. As a result, all odds ratios are less than one, meaning a reduction in the odds of bacteria relative to the reference groups.

The surface type is statistically significant in the unadjusted model (likelihood ratio test *p*-value = 0.038). The two surfaces with least bacteria, seat and uniform, significantly reduce the odds (*p* = 0.013 for both), with an estimated decrease in odds of approximately 81% compared to keyboards. Consoles had reduced odds of bacterial contamination by just over 60% (*p* = 0.085). Additionally, the estimated reduction in odds is fairly high for other surfaces compared to the reference group, although not statistically significant. The adjusted logistic regression model found that both bacteria (*p* = 0.0287) and surface type (*p* = 0.0011) were statistically significant. Keyboards were 5.8758 times more likely to have bacterial contamination than seats and uniforms, and floors were 4.1053 times more likely to be contaminated than seats and uniforms (*p* = 0.0445).

Since the different fire stations at FD2 service different community demographics, we investigated whether rates of EMS calls between the stations would influence the interpretation of surface contamination. We computed rate ratios where the rates are observed counts of bacteria in each FD2 station divided by the number of EMS calls for that station (Table 8). In other words, the rates are:

(1) $$Station\;rate\;=\;\frac{\#\;observed\;pathogen\;(out\;of\;49\;possible)}{\#\;EMS\;calls}\;=\;“pathogens/EMS”$$

We used station A as the reference group as it has the lowest rate of contamination with all five pathogens and the highest number of EMS calls (Table 8). All other stations differ from station A significantly, with rate ratios suggesting a much higher rate of observed bacteria count per EMS call. B was 5.8 times greater (*p* = 0.01, CI: 1.66, 19.88), C was 5 times greater (*p* = 0.01, CI: 1.52, 16.33), and D was 7.9 times greater (*p* = 0.0027; CI: 2.30, 27.41) (Table 9).

## 4. Discussion

Fire departments have access to standard operation protocols for exposure control of occupational health hazards, including exposure to infectious pathogens. Internal occupational exposure control protocols provide methods of compliance for work practices (e.g., washing hands with soap and water, flushing mucus membranes with water, and disposing of infectious waste), for personal protective equipment (PPE), for vaccines, and for post-exposure evaluations. External occupational exposure control protocols also provide recommendations for firefighters, specific to exposure control. For example, the CDC has recommendations for firefighters and EMS workers providing medical treatment to and transporting ill patients that are specific to infectious viruses like SARS-CoV-2 [61]. These external protocols provide PPE recommendations (e.g., wearing N95 respirators, gloves, eye protection, and gowns), PPE replacement and disposal guidelines, and recommendations for PPE and surface decontamination.

Environmental surfaces can become heavily contaminated with pathogens, leading to contact transmission and increased risks of infection [31,62,63,64]. Studies have reported that most gram-positive bacteria can survive for a few days to several months on dry surfaces. Examples include *Enterococcus* spp. (5 days to 4 months) and *Staphylococcus aureus* (7 days to 7 months) [36,65,66]. Many gram-negative bacteria, such as *Acinetobacter* spp., *E. coli*, *Klebsiella* spp., and *M. tuberculosis*, can survive for just as long [65,67,68]. Yeasts have been shown to be viable on environmental surfaces for up to 4 to 5 months [69]. Most respiratory viruses (e.g., influenza, SARS, and SARS-CoV-2) can persist on surfaces for up to a week, whereas viruses from the gastrointestinal tract (e.g., astrovirus, poliovirus, or rotavirus) are viable for about 2 months [39,69]. Further, evidence has indicated that the type of surface material plays a role in the survival time of some pathogens [70,71]. The longer a pathogen persists on a surface, the longer it may be a source of transmission and infection [69]. Thus, surface material along with disinfection agents and methods are factors in decontamination protocol development. The risk of cross-contamination poses another challenge in firefighter environments, due to firefighters who may act as vectors by moving through vehicles, stations, and other environments [2,72,73]. Effective cleaning strategies for all environments are necessary to minimize the risk of exposure. Increasing disinfection time points of surfaces, disrupting the contamination cycle, and increased hand and PPE hygiene are critical to reduce first responder risk.

It was noted during station visits that the standard operation protocols were not consistent. For instance, FD1 mopped their entryway daily, while FD2 provided disinfecting entry mats in front of entryways into the work–live space. It was noted that some fire stations were correctly using the entry mats, while others were not. It was observed that all firefighters from both departments entered the station and filed reports on the keyboards prior to washing hands. One station was so busy that firefighters went to multiple calls before returning to the station, generating reports, and then washing hands. It was also observed that handwashing practices were not consistent within or between fire departments, and none of the fire stations in this study had handwashing facilities located outside of the work–live space. Further research is warranted to investigate if these observations are contributing to environmental surface contamination and risk of firefighter exposure to HAI.

Whether microbes are from medical calls or from firefighters, they still pose a risk to both the firefighter and the patients they care for. The CDC has outlined guidelines for preventing transmission of infectious agents in healthcare settings, which include universal surveillance, contact precautions for patients identified as carriers of MRSA or other drug-resistant organisms, enhanced hand hygiene, and individual responsibility for infection control for anyone with patient contact [74,75]. Research has shown that even though MRSA is endemic, medical workers have higher rates of MRSA colonization than the general population [76]. VRE causes infection for inpatients with weakened immune systems, it has a 10% fatality rate, and it is spread by person-to-person contact or contact with contaminated surfaces [77].

*M. tuberculosis* was detected on the keyboards at two fire stations, and the washing machine at one of those stations. While tuberculosis transmission occurs primarily via respiratory droplets and not surfaces [78], laboratory experiments have shown that ingestion of *M. tuberculosis* can cause disease [79]. The presence of TB on keyboards is concerning since it is a high-touch area that all workers at the fire station are exposed to. Florida and Texas have the highest rates of tuberculosis in the United States and the fire stations we sampled are adjacent to counties with the highest transmission rates for the state [80,81,82].

The design of this environmental surface sampling study has limitations that must be considered to assign the appropriate context to its outcomes. This study was not designed to determine if the microbes detected are brought in by firefighters from active duty or from off duty and did not coordinate sampling times with the cleaning schedules of the fire stations. Additionally, the small sample size collected over a few days makes the statistical estimates less precise. Thus, the true burden of environmental contamination cannot be determined, as the data set was small with a limited number of sampling sites. Viruses were not detected on any sample in this study. The lack of detection of influenza viruses is likely due to the lack of influenza circulation in the general population at the time of sample collection. Clearly, a comprehensive environmental sampling and clinical study is needed to better understand occupational exposures of firefighters to HAI.

## 5. Conclusions

Multiple types of microbes associated with HAI were detected on surfaces at every fire station sampled and all fire stations exhibited similar incidences of bacterial contamination. A comprehensive environmental sampling and clinical study is needed to better understand occupational exposures of firefighters to HAI.

## Figures and Tables

**Table 1:** Common healthcare-associated infectious agents and their justification as targets in this study.

Organism	Classification	Justification
**Bacteria**		
*M. tuberculosis*	Gram -	Nosocomial transmission [40,41]
*E. coli*	Gram -	Contaminates surfaces and textiles [42,43]
*P. aeruginosa*	Gram -	Contaminates surfaces in ambulances [44,45]
*E. faecalis*	Gram +	Contaminates surfaces and textiles [42,43]
*C. difficile*	Gram +	Contaminates surfaces and textiles [42,43] and resistant to laundering [43]
MRSA	Gram +	Present in EMS workers and on EMS equipment [6,46]
*S. pneumoniae*	Gram +	Present in EMS workers and on EMS equipment [6,46]
**Viruses**		
Influenza	Enveloped	Highly contagious, vaccine available but many refuse [47]
Adenovirus	Non-Enveloped	Highly contagious, major cause of emergencies in elderly [48]
Norovirus	Non-Enveloped	Highly contagious, spread by contact and aerosol, environmentally stable [49,50]

**Table 2:** Primers and probes (5′-3′) used for detection of nucleic acids on surfaces.

	Forward	Reverse	Probe	Ref.
*M. tuberculosis*	CTCGACCTGAAAGACGTTATCC	CTCGGCTAGTGCA TTGTCATA	FAM-AGTACACAT/ZEN quencher/CGATCCGGTTCAAGCG-BHQ	[51]
*E. coli* O157:H7	CAACGTGGATTTCATCAA	TAGGTATATCGGAAGGAGA	FAM-AGCAACCGTTCCATTACTTACAG-BHQ	[52]
*E. faecalis*	CCCATAGTAAAGGATACATAC	CGCTGTGATTTCTTCTTA	FAM-CCTGAATGAATTGAACACCATGCCT-BHQ	[52]
*P. aeruginosa*	GGCGTGGGTGTGGAAGTC	TGGTGGCGATCTTGAACTTCTT	FAM-TGCAGTGGAACGACA-MGBNFQ	[53]
*C. difficile*	GCAAGTTGAGCGATTTACTTCGGT	GTACTGGCTCACCTTTGATATTYAAGAG	FAM-TGCCTCTCAAATATATTATCCCGTATTAG-BHQ1	[54]
MRSA	AAAGCGATTGATGGTGATACGGTT	TGCTTTGTTTCAGGTGTATCAACCA	FAM-ATGTACAAAGGTCAACCAATGACATTYAGA-BHQ	[55]
*S. pneumoniae*	TAAACAGTTTGCCTGTAGTCG	CCCGGATATCTCTTTCTGGA	FAM-AACCTTTGTTCTCTCTCGTGGCAGCTCAA-BHQ	[56]
Adenovirus	CGTCTTCAAYCGCTT	TGTAGACGTAGGGACAGG	FAM-CCGTCAGTGAAAACGTGC-BHQ	[57]
Norovirus 1	GTAAATGATGATGGCGTCTAA	ACCCADCCATTRTACATYTG	FAM-GATGGCGTCTAAGGACGC-BHQ	[58]
Norovirus 2	CTYAGGCARATGTACTGGACY	TCGACGCCATCTTCATTCAC	FAM-GGAGCCAGATTGCGATCGC-BHQ	[58]
Influenza A	CCMAGGTCGAAACGTAYGTTCTCTCTATC	TGACAGRATYGGTCTTGTCTTTAGCCAYTCCA	FAM-ATYTCGGCTTTGAGGGGGCCTG-MGB	[59]
Influenza B	GGAGCAACCAATGCCAC	GTKTAGGCGGTCTTGACCAG	FAM-ATAAACTTTGAAGCAGGAAT-MGB	[60]

**Table 3:** Pathogens found on sampling sites by week of collection (1, 2, 3, 4) at FD1.

	*M. tuberculosis*	*E. coli*	*E. faecalis*	*P. aeruginosa*	*C. difficile*	MRSA	*S. pneumoniae*
Uniform		1			1	2	
Keyboard		2, 1				1, 2, 3	2
Washer			1	1, 2, 3		1	3
Floor		3		1, 4		1, 4	1, 2
Bag		1, 3					
Console	2	2, 3					3
Seat		3					

**Table 4:** Pathogens found on sampling sites by station location (A, B, C, D) at FD2.

	*M. tuberculosis*	*E. coli*	*E. faecalis*	*P. aeruginosa*	*C. difficile*	MRSA	*S. pneumoniae*
Uniform							
Keyboard	A, C	B, C	A			C	D
Washer	C			D		D	
Floor		B	A	A			
Bag		A, D	B, C, D				
Console		C	B				
Seat			B				

**Table 5:** Significance of the incidence of bacteria on surfaces at both FD. *p*-values were obtained using Tukey–Kramer HSD adjustments for multiple comparisons to determine if one type of surface was more likely to have bacteria than another. No statistical significance was found.

	Uniform	Keyboard	Washer	Floor	Bag	Console	Seat
Uniform		0.0722	0.6177	0.4283	0.9179	0.9795	0.9999
Keyboard			0.9179	0.9795	0.6177	0.4283	0.0722
Washer				0.9999	0.9977	0.9795	0.6177
Floor					0.9795	0.9179	0.4283
Bag						0.9999	0.9179
Console							0.9795

**Table 6:** Significance of the incidence of specific bacteria at both FD. *p*-values were obtained using Tukey–Kramer HSD adjustments for multiple comparisons to determine if one type of bacteria was more likely to be present than another.

	*M. tuberculosis*	*E. coli*	*E. faecalis*	*P. aeruginosa*	*C. difficile*	MRSA	*S. pneumoniae*
*M. tuberculosis*		0.0117	0.9140	0.9783	0.9783	0.7852	0.9975
*E. coli*			0.2524	0.1368	0.0004	0.4149	0.0666
*E. faecalis*				0.9999	0.4149	0.9999	0.9975
*P. aeruginosa*					0.6056	0.9975	0.9999
*C. difficile*						0.2524	0.7852
MRSA							0.9783

**Table 7:** Logistic regression models of surface contamination and bacteria type at both FD. The unadjusted columns are for models with just the single factor (either surface or pathogen) and the adjusted columns are for a model with both included. OR = odds ratio, CI = confidence interval, and # = likelihood ratio test of the overall factor in the model.

	Unadjusted		Adjusted	
	OR (CI)	*p*-Value	OR (CI)	*p*-Value
**Surface**				
Keyboard	Reference	0.0380 ^#^	Reference	0.0011 ^#^
Floor	0.7191 (0.2856, 1.8103)	0.4839	0.6987 (0.2670, 1.8296)	0.4654
Washer	0.6334 (0.2461, 1.6300)	0.3437	0.6096 (0.2279, 1.6306)	0.3241
Bag	0.4725 (0.1728, 1.2922)	0.1441	0.4462 (0.1572, 1.2662)	0.1294
Console	0.3969 (0.1389, 1.1339)	0.0845	0.3713 (0.1254, 1.0995)	0.0736
Seat	0.1872 (0.0501, 0.6997)	0.0127	0.1702 (0.0442, 0.6555)	0.0101
Uniform	0.1872 (0.0501, 0.6997)	0.0127	0.1702 (0.0442, 0.6555)	0.0101
**Pathogen**				
*E. coli*	Reference	0.0014 ^#^	Reference	0.0287 ^#^
MRSA	0.4787 (0.1910, 1.2001)	0.1162	0.4584 (0.1781, 1.1802)	0.1060
*E. faecalis*	0.4167 (0.1617, 1.0739)	0.0699	0.3966 (0.1499, 1.0491)	0.0624
*P. aeruginosa*	0.3571 (0.1338, 0.9530)	0.0398	0.3379 (0.1235, 0.9249)	0.0347
*S. pneumoniae*	0.3000 (0.1075, 0.8372)	0.0215	0.2822 (0.0987, 0.8071)	0.0183
*M. tuberculosis*	0.1923 (0.0596, 0.6200)	0.0058	0.1788 (0.0542, 0.5893)	0.0047
*C. difficile*	0.0455 (0.0058, 0.3570)	0.0033	0.0415 (0.0052, 0.3306)	0.0026

**Table 8:** Number of calls by type of call for FD2 during 2020.

Station	Total Calls/Year	EMS	Other	Fires
**A**	7624	6302	1322	89
**B**	1501	1095	406	42
**C**	1862	1517	345	28
**D**	1106	794	312	17

**Table 9:** Logistic regression models of rate of bacterial contamination per EMS call for FD2. OR = odds ratio. CI = confidence interval.

Station	OR (CI)	*p*-Value Fisher Exact
**A**	1.00	
**B**	5.7552 (1.6661, 19.8797)	0.0094
**C**	4.9851 (1.5214, 16.3343)	0.01
**D**	7.937 (2.2977, 27.416)	0.0027

## Data Availability

All relevant data are included in the manuscript.

## References

[1] National Fire Protection Association Fire Experience Survey: Fire Department Calls. https://www.nfpa.org/News-and-Research/Data-research-and-tools/Emergency-Responders/Fire-department-calls.

[2] Boal W.L., Hales T., Ross C.S. (2005). Blood-Borne Pathogens among Firefighters and Emergency Medical Technicians. Prehospital Emerg. Care.

[3] Sepkowitz K.A., Eisenberg L. (2005). Occupational Deaths among Healthcare Workers. Emerg. Infect. Dis..

[4] Le A.B., Buehler S.A., Maniscalco P.M., Lane P., Rupp L.E., Ernest E., Von Seggern D., West K., Herstein J.J., Jelden K.C. (2018). Determining training and education needs pertaining to highly infectious disease preparedness and response: A gap analysis survey of US emergency medical services practitioners. Am. J. Infect. Control..

[5] Bitely C., Miller B., Glauser J. (2019). EMS Disease Exposure, Transmission, and Prevention: A Review Article. Curr. Emerg. Hosp. Med. Rep..

[6] Orellana R.C., Hoet A.E., Bell C., Kelley C., Lu B., Anderson S.E., Stevenson K.B. (2016). Methicillin-resistant Staphylococcus aureus in Ohio EMS Providers: A Statewide Cross-sectional Study. Prehospital Emerg. Care.

[7] Magill S.S., Edwards J.R., Bamberg W., Beldavs Z.G., Dumyati G., Kainer M.A., Lynfield R., Maloney M., McAllister-Hollod L., Nadle J. (2014). Multistate Point-Prevalence Survey of Health Care–Associated Infections. N. Engl. J. Med..

[8] Pang Z., Raudonis R., Glick B.R., Lin T.-J., Cheng Z. (2019). Antibiotic resistance in Pseudomonas aeruginosa: Mechanisms and alternative therapeutic strategies. Biotechnol. Adv..

[9] Miller W., Munita J.M., Arias C. (2014). Mechanisms of antibiotic resistance in enterococci. Expert Rev. Anti-infective Ther..

[10] Cillóniz C., Garcia-Vidal C., Ceccato A., Torres A., Fong I.W., Shlaes D., Drlica K. (2018). Antimicrobial Resistance Among Streptococcus pneumoniae. Antimicrobial Resistance in the 21st Century.

[11] Karlsson E.A., Mook P.A.N., Vandemaele K., Fitzner J., Hammond A., Cozza V., Zhang W., Moen A. (2021). Review of global influenza circulation, late 2019 to 2020, and the impact of the COVID-19 pandemic on influenza circulation. Wkly. Epidemiol. Rec..

[12] Dini G., Toletone A., Sticchi L., Orsi A., Bragazzi N.L., Durando P. (2018). Influenza vaccination in healthcare workers: A comprehensive critical appraisal of the literature. Hum. Vaccines Immunother..

[13] To K., Lai A., Lee K., Koh D., Lee S. (2016). Increasing the coverage of influenza vaccination in healthcare workers: Review of challenges and solutions. J. Hosp. Infect..

[14] National Fire Protection Association (2018). NFPA 1582, Standard on Comprehensive Occupational Medical Program for Fire Departments.

[15] Engel R. (2021). Will you get Vaccinated for COVID-19? Firefighters Weigh in. Fire Rescue 1, 02/02/2021 ed..

[16] Beadling C., Slifka M.K. (2004). How do viral infections predispose patients to bacterial infections?. Curr. Opin. Infect. Dis..

[17] Hanada S., Pirzadeh M., Carver K.Y., Deng J.C. (2018). Respiratory Viral Infection-Induced Microbiome Alterations and Secondary Bacterial Pneumonia. Front. Immunol..

[18] Yildiz S., Mazel-Sanchez B., Kandasamy M., Manicassamy B., Schmolke M. (2018). Influenza A virus infection impacts systemic microbiota dynamics and causes quantitative enteric dysbiosis. Microbiome.

[19] Gaibani P., Viciani E., Bartoletti M., Lewis R.E., Tonetti T., Lombardo D., Castagnetti A., Bovo F., Horna C.S., Ranieri M. (2021). The lower respiratory tract microbiome of critically ill patients with COVID-19. Sci. Rep..

[20] Wobus C., Niendorf S., Taube S., Hoehne M., Young V.B., Rogers M.A., Stokely J.N. (2016). Prevalence of human norovirus and *Clostridium difficile* coinfections in adult hospitalized patients. Clin. Epidemiol..

[21] Shuai H., Bian Q., Luo Y., Zhou X., Song X., Ye J., Huang Q., Peng Z., Wu J., Jiang J. (2020). Molecular characteristics of Clostridium difficile in children with acute gastroenteritis from Zhejiang. BMC Infect. Dis..

[22] Teter J., Millin M.G., Bissell R. (2014). Hand Hygiene in Emergency Medical Services. Prehospital Emerg. Care.

[23] Valdez M.K., Sexton J.D., Lutz E.A., Reynolds K.A. (2015). Spread of infectious microbes during emergency medical response. Am. J. Infect. Control..

[24] Barr N., Holmes M., Roiko A., Dunn P., Lord B. (2017). Self-reported behaviors and perceptions of Australian paramedics in relation to hand hygiene and gloving practices in paramedic-led health care. Am. J. Infect. Control..

[25] Vikke H.S., Vittinghus S., Betzer M., Giebner M., Kolmos H.J., Smith K., Castrén M., Lindström V., Mäkinen M., Harve H. (2019). Hand hygiene perception and self-reported hand hygiene compliance among emergency medical service providers: A Danish survey. Scand. J. Trauma, Resusc. Emerg. Med..

[26] Vikke H.S., Vittinghus S., Giebner M., Kolmos H.J., Smith K., Castrén M., Lindström V. (2019). Compliance with hand hygiene in emergency medical services: An international observational study. Emerg. Med. J..

[27] Oh H.S., Uhm D. (2016). Occupational exposure to infection risk and use of personal protective equipment by emergency medical personnel in the Republic of Korea. Am. J. Infect. Control..

[28] Barr N., Holmes M., Roiko A., Dunn P., Lord B. (2018). Challenges for environmental hygiene practices in Australian paramedic-led health care: A brief report. Am. J. Infect. Control..

[29] Wepler M., Stahl W., Von Baum H., Wildermuth S., Dirks B., Georgieff M., Hafner S. (2015). Prevalence of nosocomial pathogens in German ambulances: The SEKURE study. Emerg. Med. J..

[30] Carling P.C., Perkins J., Ferguson J., Thomasser A. (2014). Evaluating a New Paradigm for Comparing Surface Disinfection in Clinical Practice. Infect. Control. Hosp. Epidemiology.

[31] Casanova L., Sobsey M.D., Pfaender F., Rutala W., Vinje J., Weber D. (2008). Survival and Transmission of Coronaviruses in the Healthcare Environment.

[32] Cheng V.C., Wong S.C., Chan V.W., So S.Y., Chen J.H., Yip C.C., Chan K.H., Chu H., Chung T.W., Sridhar S. (2020). Air and environmental sampling for SARS-CoV-2 around hospitalized patients with coronavirus disease 2019 (COVID-19). Infect. Control. Hosp Epidemiol..

[33] Cobrado L., Silva-Dias A., Azevedo M.M., Rodrigues A.G. (2017). High-touch surfaces: Microbial neighbors at hand. Eur. J. Clin. Microbiol. Infect. Dis..

[34] Donskey C.J. (2013). Does improving surface cleaning and disinfection reduce health care-associated infections?. Am. J. Infect. Control..

[35] Eckstein B., Adams D., Eckstein E., Rao A., Sethi A., Yadavalli G., Donskey C. (2007). Reduction of Clostridium Difficile and vancomycin-resistant Enterococcus contamination of environmental surfaces after an intervention to improve cleaning methods. BMC Infect. Dis..

[36] Farcas D., Blachere F.M., Kashon M.L., Sbarra D., Schwegler-Berry D., Stull J.O., Noti J.D. (2019). Survival of Staphylococcus aureus on the outer shell of fire fighter turnout gear after sanitation in a commercial washer/extractor. J. Occup. Med. Toxicol..

[37] Frickmann H., Bachert S., Warnke P., Podbielski A. (2018). Validated measurements of microbial loads on environmental surfaces in intensive care units before and after disinfecting cleaning. J. Appl. Microbiol..

[38] Gavaldà L., Pequeño S., Soriano A., Domínguez M.A. (2015). Environmental contamination by multidrug-resistant microorganisms after daily cleaning. Am. J. Infect. Control..

[39] Howie R., Alfa M., Coombs K. (2008). Survival of enveloped and non-enveloped viruses on surfaces compared with other micro-organisms and impact of suboptimal disinfectant exposure. J. Hosp. Infect..

[40] Bouvet E. (2001). Nosocomial pulmonary tuberculosis. Rev. de Pneumol. Clin..

[41] Seto J., Otani Y., Wada T., Suzuki Y., Ikeda T., Araki K., Mizuta K., Ahiko T. (2019). Nosocomial Mycobacterium tuberculosis transmission by brief casual contact identified using comparative genomics. J. Hosp. Infect..

[42] El-Mokhtar M.A., Hetta H. (2018). Ambulance vehicles as a source of multidrug-resistant infections: A multicenter study in Assiut City, Egypt. Infect. Drug Resist..

[43] Mackay W., Whitehead S., Purdue N., Smith M., Redhead N., Williams C., Wilson S. (2017). Infection control implications of the laundering of ambulance staff uniforms and reusable mops. J. Hosp. Infect..

[44] Cameron J.L., Reese W.A., Tayal V.S., Clark R.F., Kelso D., Gonzalez E.R., Garnett A.R., Ornalo J.P. (1986). Bacterial contamination of ambulance oxygen humidifier water reservoirs: A potential source of pulmonary infection. Ann. Emerg. Med..

[45] Kober P., Labes H., Möller H., Hülsse C., Kramer A. (2001). Hygienestatus von Fahrzeugen und Ausrüstung im Rettungsdienst. AINS-Anästhesiologie · Intensivmedizin · Notfallmedizin · Schmerztherapie.

[46] Gibson C.V. (2018). Emergency medical services oxygen equipment: A fomite for transmission of MRSA?. Emerg. Med. J..

[47] Moser A., Mabire C., Hugli O., Dorribo V., Zanetti G., Lazor-Blanchet C., Carron P.-N. (2016). Vaccination Against Seasonal or Pandemic Influenza in Emergency Medical Services. Prehospital Disaster Med..

[48] Monge S., Duijster J., Kommer G.J., Van De Kassteele J., Krafft T., Engelen P., Valk J.P., De Waard J., De Nooij J., Riezebos-Brilman A. (2020). Ambulance dispatch calls attributable to influenza A and other common respiratory viruses in the Netherlands (2014–2016). Influ. Other Respir. Viruses.

[49] Bhatta M.R., Marsh Z., Newman K.L., Rebolledo P.A., Huey M., Hall A.J., Leon J.S. (2020). Norovirus outbreaks on college and university campuses. J. Am. Coll. Health.

[50] Rajagopalan S., Yoshikawa T.T. (2016). Norovirus Infections in Long-Term Care Facilities. J. Am. Geriatr. Soc..

[51] Wang H.-Y., Lu J.-J., Chang C.-Y., Chou W.-P., Hsieh J.C.-H., Lin C.-R., Wu M.-H. (2019). Development of a high sensitivity TaqMan-based PCR assay for the specific detection of Mycobacterium tuberculosis complex in both pulmonary and extrapulmonary specimens. Sci. Rep..

[52] Liu Y., Cao Y., Wang T., Dong Q., Li J., Niu C. (2019). Detection of 12 Common Food-Borne Bacterial Pathogens by TaqMan Real-Time PCR Using a Single Set of Reaction Conditions. Front. Microbiol..

[53] Lee C.S., Wetzel K., Buckley T., Wozniak D., Lee J. (2011). Rapid and sensitive detection of Pseudomonas aeruginosa in chlorinated water and aerosols targeting gyrB gene using real-time PCR. J. Appl. Microbiol..

[54] Muñoz M., Ríos-Chaparro D.I., Herrera G., De León S.C.S., Birchenall C., Pinilla D., Pardo-Oviedo J.M., Josa D.F., Patarroyo M.A., Ramírez J.D. (2018). New Insights into Clostridium difficile (CD) Infection in Latin America: Novel Description of Toxigenic Profiles of Diarrhea-Associated to CD in Bogotá, Colombia. Front. Microbiol..

[55] Wang H.-Y., Kim S., Kim J., Park S.-D., Uh Y., Lee H. (2014). Multiplex Real-Time PCR Assay for Rapid Detection of Methicillin-Resistant Staphylococci Directly from Positive Blood Cultures. J. Clin. Microbiol..

[56] Tavares D.A., Handem S., Carvalho R.J., Paulo A.C., De Lencastre H., Hinds J., Sá-Leão R. (2019). Identification of Streptococcus pneumoniae by a real-time PCR assay targeting SP2020. Sci. Rep..

[57] Buckwalter S.P., Teo R., Espy M.J., Sloan L.M., Smith T.F., Pritt B.S. (2011). Real-Time Qualitative PCR for 57 Human Adenovirus Types from Multiple Specimen Sources. J. Clin. Microbiol..

[58] Kong B.-H., Lee S.-G., Han S.-H., Jin J.-Y., Jheong W.-H., Paik S.-Y. (2015). Development of Enhanced Primer Sets for Detection of Norovirus. BioMed Res. Int..

[59] World Health Organization (2012). WHO Information for Molecular Diagnosis of Influenza Virus in Humans.

[60] World Health Organization (2011). WHO Information for Molecular Diagnosis of Influenza Virus in Humans—Update.

[61] Centers for Disease Control and Prevention (2021). What Firefighters and EMS Providers Need to Know about COVID-19.

[62] Weber D.J., Anderson D., Rutala W.A. (2013). The role of the surface environment in healthcare-associated infections. Curr. Opin. Infect. Dis..

[63] Weber D.J., Rutala W.A., Miller M.B., Huslage K., Sickbert-Bennett E. (2010). Role of hospital surfaces in the transmission of emerging health care-associated pathogens: Norovirus, Clostridium difficile, and Acinetobacter species. Am. J. Infect. Control.

[64] Roberts M.C., No D.B. (2014). Environment surface sampling in 33 Washington State fire stations for methicillin-resistant and methicillin-susceptible Staphylococcus aureus. Am. J. Infect. Control.

[65] Neely A.N., Maley M.P. (2000). Survival of Enterococci and Staphylococci on Hospital Fabrics and Plastic. J. Clin. Microbiol..

[66] Almatroudi A., Gosbell I., Hu H., Jensen S., Espedido B., Tahir S., Glasbey T., Legge P., Whiteley G., Deva A. (2016). Staphylococcus aureus dry-surface biofilms are not killed by sodium hypochlorite: Implications for infection control. J. Hosp. Infect..

[67] Denton M., Wilcox M., Parnell P., Green D., Keer V., Hawkey P., Evans I., Murphy P. (2004). Role of environmental cleaning in controlling an outbreak of Acinetobacter baumannii on a neurosurgical intensive care unit. J. Hosp. Infect..

[68] Weaver L., Michels H., Keevil C. (2008). Survival of Clostridium difficile on copper and steel: Futuristic options for hospital hygiene. J. Hosp. Infect..

[69] Kramer A., Schwebke I., Kampf G. (2006). How long do nosocomial pathogens persist on inanimate surfaces? A systematic review. BMC Infect. Dis..

[70] Harris D., Taylor K.P., Napierkowski K., Zechmann B. (2021). Indoor Finish Material Influence on Contamination, Transmission, and Eradication of Methicillin-Resistant Staphylococcus aureus (MRSA). HERD Health Environ. Res. Des. J..

[71] Ronca S.E., Sturdivant R.X., Barr K.L., Harris D. (2021). SARS-CoV-2 viability on 16 common indoor surface finish materials. HERD Health Env. Res. Des. J..

[72] Lei H., Jones R.M., Li Y. (2017). Exploring surface cleaning strategies in hospital to prevent contact transmission of methicillin-resistant Staphylococcus aureus. BMC Infect. Dis..

[73] Albrich W.C., Harbarth S. (2008). Health-care workers: Source, vector, or victim of MRSA?. Lancet Infect. Dis..

[74] Jain R., Kralovic S.M., Evans M.E., Ambrose M., Simbartl L.A., Obrosky D.S., Render M.L., Freyberg R.W., Jernigan J.A., Muder R.R. (2011). Veterans Affairs Initiative to Prevent Methicillin-Resistant Staphylococcus aureus Infections. N. Engl. J. Med..

[75] Siegel J., Rhinehart E., Jackson M., Chiarello L., Control C.F.D. (2019). Healthcare Infection Control Practices Advisory Committee. 2007 Guideline for Isolation Precautions: Preventing Transmission of Infectious Agents in Healthcare Settings.

[76] Miramonti C., Rinkle J., Iden S., Lincoln J., Huffman G., Riddell E., Kozak M.A. (2012). The Prevalence of Methicillin-Resistant Staphylococcus Aureus among Out-of-Hospital Care Providers and Emergency Medical Technician Students. Prehospital Emerg. Care.

[77] National Center for HIV/AIDS, Viral Hepatitis, STD, and TB Prevention Division of Tuberculosis Elimination (2019). Vancomycin-Resistant Enterococci (VRE) in Healthcare Settings.

[78] National Center for HIV/AIDS, Viral Hepatitis, STD, and TB Prevention Division of Tuberculosis Elimination (2013). Core Curriculum on Tuberculosis: What the Clinician Should Know.

[79] Ghodbane R., Medie F.M., Lepidi H., Nappez C., Drancourt M. (2014). Long-term survival of tuberculosis complex mycobacteria in soil. Microbiology.

[80] Florida Department of Health (2020). Tuberculosis Cases 2016–2020.

[81] Texas Department of Health and Human Services (2020). Texas 2019 TB Surveillance Report.

[82] Shrestha S., Cherng S., Hill A.N., Reynolds S., Flood J., Barry P.M., Readhead A., Oxtoby M., Lauzardo M., Privett T. (2019). Impact and Effectiveness of State-Level Tuberculosis Interventions in California, Florida, New York, and Texas: A Model-Based Analysis. Am. J. Epidemiol..

